# Comparing performance between log-binomial and robust Poisson regression models for estimating risk ratios under model misspecification

**DOI:** 10.1186/s12874-018-0519-5

**Published:** 2018-06-22

**Authors:** Wansu Chen, Lei Qian, Jiaxiao Shi, Meredith Franklin

**Affiliations:** 1 Kaiser Permanente Southern California, Department of Research and Evaluation, 100 S. Los Robles Ave, 2nd Floor, Pasadena, CA 91101 USA; 2 Department of Preventive Medicine, Keck School of Medicine, University of Southern California, 1975 Zonal Ave, Los Angeles, CA 90033 USA

**Keywords:** Log-binomial regression, Robust (modified) Poisson regression, Model misspecification, Risk ratio, Link function misspecification

## Abstract

**Background:**

Log-binomial and robust (modified) Poisson regression models are popular approaches to estimate risk ratios for binary response variables. Previous studies have shown that comparatively they produce similar point estimates and standard errors. However, their performance under model misspecification is poorly understood.

**Methods:**

In this simulation study, the statistical performance of the two models was compared when the log link function was misspecified or the response depended on predictors through a non-linear relationship (i.e. truncated response).

**Results:**

Point estimates from log-binomial models were biased when the link function was misspecified or when the probability distribution of the response variable was truncated at the right tail. The percentage of truncated observations was positively associated with the presence of bias, and the bias was larger if the observations came from a population with a lower response rate given that the other parameters being examined were fixed. In contrast, point estimates from the robust Poisson models were unbiased.

**Conclusion:**

Under model misspecification, the robust Poisson model was generally preferable because it provided unbiased estimates of risk ratios.

**Electronic supplementary material:**

The online version of this article (10.1186/s12874-018-0519-5) contains supplementary material, which is available to authorized users.

## Background

### Introduction

Logistic regression is the most widely-used modeling approach for studying associations between exposures and binary outcomes. For rare events, the odds ratio estimated from logistic regression approximates the risk ratio (RR). However, when events are common, odds ratios always overestimate risk ratios [1] Zhang and Yu [2] suggested a correction for odds ratios to give a risk ratio in studies of common outcomes. This method was subsequently shown to result in inconsistent point estimates as well as invalid confidence intervals [3]. Efforts were made to modify the data so that estimated odds ratios from a logistic regression analysis were comparable to risk ratios [4]. However, it was found that these methods produced prevalence greater than one [5]. Greenland [6] brought extensive literature on valid model-based estimation of relative risks and other measures to readers’ attention. Of the model-based approaches, log-binomial [5, 7, 8] and robust (or modified) Poisson regression models [7, 9] are most frequently applied to estimate risk ratios for common binary outcomes. Barros et al. [7] and Zou [9] showed how risk ratios can be estimated by using robust Poisson regression with a robust error variance.

In medical and public health research, log-binomial and robust Poisson regression models are widely used to directly estimate risk ratios for both common and rare outcomes. For example, they can be used to estimate the effect of clinical characteristics (e.g. obesity, smoking, history of stroke, exercise, or diet) on a health condition (e.g. a cardiovascular event, mortality, or hospital admission). Our own Medline search of articles published between 2005 and 2014 demonstrated that the popularity of both models has increased significantly in the past decade (Additional file 1).

Model misspecification can lead to biased estimates, resulting in erroneous and misleading conclusions. Regression models require that the relationship between the response and the explanatory variables conforms to a particular functional form. Omitting important explanatory variables, failing to account for non-linear components or critical interaction terms, or making measurement errors can cause model misspecification and thus bias the parameter estimators of one or more of the predictors in a regression model. Under correct model specification, the log-binomial and robust Poisson methods have been shown to yield comparable point estimates and standard errors [7, 9–12]. For instance, in a simulation study with sample size of 1000, both the log-binomial and robust Poisson models yielded similar unbiased estimates with good coverage probability [12]. However, the results of the following example revealed a different paradigm.

### A motivating example

A cross-sectional study was conducted at Kaiser Permanente Southern California to assess the association of fractional exhaled nitric oxide (FeNO), a marker of airway inflammation, and asthma burden among persistent asthma patients who were treated with inhaled corticosteroids (ICS). It was hypothesized that high FeNO levels were associated with greater asthma burden. In this study, the primary binary outcome of interest was whether seven or more short-acting beta-agonist (SABA) canisters were dispensed in the past 12 months, which is a validated administrative data surrogate for asthma impairment. Previous studies based on similar patient populations revealed that the prevalence of asthma impairment among persistent asthma patients was high [13]. The study population consisted of asthma patients 12 to 56 years of age who had aeroallergen sensitization and regular ICS treatment for at least one month were enrolled during an allergy department visit (index visit). Information on patient demographics, FeNO, forced expiratory volume in one second (FEV_1_% predicted) and asthma control test (ACT) score was collected during the index visit, while the information on aeroallergen sensitization and SABA canisters dispensed came from electronic medical records.

FeNO was categorized into four quartiles. Multivariable log-binomial and robust Poisson regression models were applied to estimate the risk ratio of having seven or more SABA canisters in each of the FeNO quartiles (using the lowest quartile as the reference group), controlling for age, gender, race/ethnicity, number of aeroallergen sensitivities, clinical center, FEV_1_% predicted (≥80% vs. < 80%) and ACT score (> 19, 16–19, < 16). Age remained as a continuous variable in the model.

Based on the robust Poisson model, the risk ratio of having ≥7 SABA canisters over the past 12 months was 2.05 (95% CI, 1.03–4.05) in patients whose FeNO value was in the second quartile, compared to patients whose FeNO value was in the first quartile (Additional file 2). However, when the analysis was repeated by using the log-binomial model, the corresponding risk ratio was 1.67 (95% CI, 0.83–3.57), a 19% reduction from the robust Poisson estimate. Although the point estimates for FeNO from the two models were in the same direction, the interpretation of the results varied because the 95% confidence interval of the estimated RR from the robust Poisson covered one, yet the log-binomial model did not. The RR for patients whose FeNO values were in the third and fourth quartiles compared to the patients in the first quartile were also different between the two models, although the interpretations remained the same at the 95% level (see Additional file 2). The differences in point estimates of RR between the two models were in the range of 12–19%.

### Current study

The inconsistent conclusions between the aforementioned simulation studies [7, 9–12] and the results from the motivating example led us to further investigate whether model misspecification and/or characteristics of the data resulted in such large discrepancies between log-binomial and the robust Poisson regression models.

An initial attempt was made to understand the impact of model misspecification on the performance of the two regression models when an important explanatory variable was omitted, a higher order term of non-linear explanatory variable was ignored, or an interaction term was overlooked (Kaiser Permanente, Pasadena; University of Southern California, Los Angeles; unpublished results. Additional file 3). Although certain types of model misspecification did bias the point estimates, both the magnitude and the direction of the biases were comparable between the two models. The current simulation study was subsequently designed to examine the impact of misspecified link functions and truncated probabilities, one type of linear predictor misspecification. To our knowledge, the extent of such an inconsistency between the two models has not been systematically examined. Inspired by the differences in the estimating equations of the two models, we also examined the impact of having observations with large probabilities of developing the response on the performance of the two models when the overall response rate varies.

The paper is organized as follows: The “Methods” section presents the theory behind the two models to explain the differences in the estimation methods that could result in variations in the estimates. The "Methods" section also provides the details of the simulation design. The "Results" section shows the results of simulation under various scenarios. Lastly, we summarize the findings and provide recommendations for the use of these models in future studies in the “Discussion” section.

## Methods

Generalized linear models (GLM) originate from a significant extension of traditional linear regression models [14]. They consist of a random component that specifies the conditional distribution of the response variable (*Y*) from an exponential family given the values of the explanatory variables *X*_1,_*X*_2,_···,*X*_k_, a linear predictor (or systematic) component that is a linear function of the predictors, *ƞ*=*β*_0_+*β*_1_*X*_1_+*β*_2_*X*_2_+···+*β*_*k*_*X*_*k*_, where *β=*(*β*_0_,*β*_1_,...,*β*_*k*_)^*T*^ is the vector of the parameters*,* and a smooth invertible link function that transforms the expectation of the response variable, *μ* ≡ *E(Y*), to the linear predictors: *g*(*μ*)=*ƞ=β*_0_+*β*_1_X_1_+*β*_2_X_2_+···*β*_*k*_*X*_*k*_. For example, the most common link for binary outcomes is the logit (i.e. log (*μ*/(1-*μ*))) in a logistic model, the log (*μ*) in a Poisson model, or a log-binomial model.

In the descriptions below, *Y*_*i*_ and ${x}_i^T=\left(1,{x}_{i1},{x}_{i2},\dots, {x}_{ik}\right)$ denote the binary outcome and the row vector comprised of *k* predictors for the i^th^ individual (i = 1,2,…n), respectively. The observations from the *n* individuals are independent.

### Log-binomial regression

In the GLM framework, the conditional distribution of *Y*_*i*_ given the predictor variables is binomial, with the mean response related to the predictors by the link function log (*μ*_*i*_). In log-binomial regression, *μ*_*i*_ is often denoted as *p*_*i*_, because *E(Y*_*i*_) is a probability with a value between zero and one. Although there are other methods to obtain efficient estimators, the maximum likelihood approach is used to generate asymptotically efficient estimators (maximum likelihood estimates (MLE)) in log-binomial regressions [5, 8].

The MLE of log-binomial models are derived from an iteratively reweighted least squares (IRLS) approach [15]. In a log-binomial regression, $\log \left({P}_i\left(\beta \right)\right)={x}_i^T\beta$ where *p*_*i*_(*β*)=Pr(*y*_*i*_=1|*x*_*i*_),0 ≤ *p*_*i*_ ≤ 1, and ${x}_i^X\beta <0$ (constrained). The log-likelihood is given by

1 $$ \mathrm{\ell}\left(\beta \right)=\sum \limits_{i=1}^n{y}_i\log \left({p}_i\left(\beta \right)\right)+\sum \limits_{i=1}^n\left(1-{y}_i\right)\log \left(1-{p}_i\left(\beta \right)\right)\cdot $$

It can be proven that the MLE for *β* can be found by the following iteration (Additional file 4)

2 $$ {\beta}^{\left(t+1\right)}={\left({X}^{\hbox{'}} WX\right)}^{-1}\left({X}^{\hbox{'}} Wz\right) $$

$$ \mathrm{where}\kern0.28em z=X{\beta}^{(t)}+\frac{\left(Y-P\left({\beta}^{(t)}\right)\right)}{P\left({\beta}^{(t)}\right)},X=\left({x}_{i,j}\right)\in {R}^{n,k},\frac{Y-P\left(\beta \right)}{P\left(\beta \right)}={\left(\frac{y_1-{p}_1\left(\beta \right)}{p_1\left(\beta \right)},\frac{y_2-{p}_2\left(\beta \right)}{p_2\left(\beta \right)},\dots, \frac{y_n-{p}_n\left(\beta \right)}{p_n\left(\beta \right)}\right)}^T,\mathrm{and}\kern0.34em \mathrm{weight}\kern0.28em \mathrm{W}= Diag\left(\frac{p_i\left(\beta \right)}{1-{p}_i\left(\beta \right)}\right),\mathrm{i}=1,2,\dots, \mathrm{n};\mathrm{j}=1,2,\dots, \mathrm{k}. $$

The iteration process continues until *β* stabilizes. The weights W used in the iterative process contain *p*_*i*_(*β*) in the numerator and 1 – *p*_*i*_(*β*) in the denominator, where *p*_*i*_(*β*) = exp (${x}_i^T\beta$) with a range from 0 to 1. When *p*_*i*_(*β*) is a very small number, the weight approximates *p*_*i*_(*β*). When *p*_*i*_(*β*) approaches one, the weight approaches infinity. This suggested that the IRLS approach is highly influenced by observations that have large *p*_*i*_(*β*). Moreover, the impact is also influenced by the average *p*_*i*_(*β*), or the average weight (lower average *p*_*i*_(*β*) is associated with lower average weight). For illustration, we constructed two hypothetical samples each with five observations having the following probabilities: sample 1 = {0.1, 0.3, 0.4, 0.5, 0.95} and sample 2 = {0.02, 0.03, 0.08, 0.15, 0.95}. The corresponding weights for the two samples were {0.25, 0.43, 0.67, 1, 19} and {0.02, 0.03, 0.09, 0.18, 19}, respectively. In sample 2, the observation with weight 19 will impact the point estimate more, compared the observation in sample 1 with the same weight.

### Robust Poisson regression

In robust Poisson regression, a quasi-likelihood (QL) model can be applied to fit the data with a binary outcome [14–18]. Quasi-likelihood was first introduced by Wedderburn (1974) as a function that has properties analog to those of log-likelihood functions [18]. Similar to ML method, maximum QL method can be used to estimate the QL estimates. In a maximum QL model, only the relationship between the mean and the variance (i.e. the variance is a function of the mean) needs to be specified instead of the underlying distribution of the data [15–19]. It can be shown that when *Y*_*i*_ comes from the exponential family, the quasi-score function is identical to the score function associated with the maximum likelihood of the GLM.

When the Poisson distribution is chosen, the quasi-score function can be simplified to ${S}_j\left(\beta \right)=\frac{1}{\phi}\sum \limits_{i=1}^n\left({y}_i-{\mu}_i\right){x}_{ij}$, resulting in quasi-score estimating equations ${S}_j\left(\beta \right)=\sum \limits_{i=1}^n\left({y}_i-{\mu}_i\right){x}_{ij}=0$, which is the same as the estimating equations of the Poisson regression models. In the two equations above, *ϕ* is the dispersion parameter, and j = 1,2,…k. The final estimate from the quasi-scoring procedure satisfies the condition ${S}_j\left(\widehat{\beta}\right)=0$ and $\widehat{\beta}$ is a consistent and asymptotically unbiased estimate of *β* [20]. $\widehat{\beta}$ does not depend on *ϕ*.

The quasi-likelihood estimators are not maximally and asymptotically efficient [14]. The robust Poisson regression model uses the classical sandwich estimator under the generalized estimation equation (GEE) framework to provide accurate standard errors for the elements [19–21]. The variance-covariance matrix is

3 $$ {\left[\sum \limits_{i=1}^nE\left[{I}_i\left(\beta \right)\right]\right]}^{-1}{\left[\sum \limits_{i=1}^nE\left[\Big({S}_i\left(\beta \right){S}_i{\left(\beta \right)}^T\right]\right]}^{-1}{\left[\sum \limits_{i=1}^nE\left[{I}_i\left(\beta \right)\right]\right]}^{-1} $$

where ${I}_i\left(\beta \right)=-\frac{\partial {S}_i\left(\beta \right)}{\partial \beta }$ is the information matrix [22]. A consistent estimate of the variance can be obtained by evaluating the variance-covariance matrix at $\widehat{\beta}$.

### Implementation

Both regression models were implemented in SAS [23] (SAS *Software Version* 9.3 of the SAS System for Unix. Cary, NC. SAS Institute Inc. 2011). The SAS codes can be found in Additional file 5. For the log-binomial model, − 4 was set as the initial value of the intercept. For both models, the weighted least squares estimates (default) were used as initial values of parameters. The convergence criterion was 10^− 4^ (default). A well-known issue of log-binomial models is failure to converge when the MLE is located on the boundary of the parameter space (i.e. the predicted probability of the outcome is equal to 1). To minimize the convergence issue, the COPY method was applied [24, 25] in which the number of virtual copies was set to 10,000. To ensure a fair comparison between the log-binomial and robust Poisson models, the evaluation was conducted by only using the results based on exactly the same simulated data. If the COPY method did not converge for a dataset, the same dataset was then removed before the performance of the robust Poison models was evaluated. The exclusion of datasets was very rare in this study. Details on the number of excluded datasets can be found in the “Discussion” section.

### Measures of model performance

For each simulated scenario, the simulation process was repeated 1000 times. In each of the 1000 simulated datasets, the log risk ratio was estimated from the log-binomial model and the robust Poisson model, respectively. For each scenario, the relative bias, standard error (SE), and mean square error (MSE) in log scale for all three measures were calculated by summarizing the results from the 1000 datasets for each regression model. Relative bias was defined as the average of the 1000 estimated *RR* in log scale minus the log of the true *RR* divided by the log of the true *RR*. For ${\widehat{\theta}}_m$, the estimated log *RR* from the m^th^ dataset using either the log-binomial model or the robust Poisson model, the relative bias was defined as $\left(\frac{1}{1,000},\sum \limits_{m=1}^{1,000},\frac{{\hat{\theta}}_m-\log (trueRR)}{\log (trueRR)}\right)\times 100\%$. Standard error was defined as the empirical SE of the estimated risk ratio in log scale over all 1000 simulations. The MSE was calculated by taking the sum of the squared bias in log scale and the variances, in which the bias was specified as $\frac{1}{1,000}\sum \limits_{m=1}^{1,000}{\hat{\theta}}_m-\log (trueRR)$.

Because both SE and MSE depended on the sample size, the process described above was repeated for sample of size 500 for all scenarios with RR = 3.

### Simulated datasets

Let *Y* be a common binary outcome (*Y* = 1 for disease and *Y* = 0 for non-disease) and *X* be a binary exposure variable (*X* = 1 for exposure and *X* = 0 for non-exposure). First, uncorrelated random variables *Z*_1_ and *Z*_2_ following the Bernoulli (0.5) and the Uniform [0, 1] distributions, respectively, were generated for 1000 subjects. These distributions were chosen for their simplicity in the design. Then, the exposure variable *X* based on the subject-specific probability of exposure, defined by the equation logit (*P* (*X* = 1| *Z*_1_, *Z*_2_)) = − 1.0 + *Z*_1_ + *Z*_2_ with E(*P*(*X* = 1| *Z*_1_, *Z*_2_)) = 0.5, was created for each subject. All of the outcome variables defined below were conditional on the exposure status and the covariates. For exposed subjects, *P* (*Y* = 1| *X* = 1, *Z*_1_, *Z*_2_) = 3 × *P* (*Y* = 1| *X* = 0, *Z*_1_, *Z*_2_). The adjusted *RR* (i.e. *P* (*Y* = 1| *X* = 1, *Z*_1_, *Z*_2_)) / *P* (*Y* = 1| *X* = 0, *Z*_1_, *Z*_2_)) was fixed at 3.0, chosen to reflect the effect size commonly seen in real-world settings, and strong enough to yield observable differences in performance between the two regression models.

#### Scenarios to study the impact of truncation

The equations to generate *Y* took four different forms (*Y*_*1*_, *Y*_2_, *Y*_3_, *Y*_4_) to enable the examination of the impact of truncation. Unlike *Y*_1_, which always had a perfect linear association with its predictors (i.e. not truncated), *Y*_2_, *Y*_3,_ and *Y*_4_ were truncated such that the values of *P* (*Y*_*k*_ = 1| *X* = 0, *Z*_1_, *Z*_2_) depended on whether or not “*Z*_1_ + (beta of Z_2_) × *Z*_2_” reached a threshold (*k* = 2, 3, and 4) (Table 1). For example, in Scenario I-2, the threshold was set at 0.15, requiring “*Z*_1_ + 3 × *Z*_2_” to be greater than 0.15 to impact *P* (*Y*_*2*_ = 1| *X* = 0, *Z*_1_, *Z*_2_)). The defined threshold varied by scenario and was chosen such that the percentages of exposed subjects at the maximum *P* (*Y* = 1| *X*, *Z*_1_, *Z*_2_)) could be controlled within the range of 1.4–5.8%. A truncation yielded a spike of observations at the maximum *P* (*Y* = 1| *X*, *Z*_1_, *Z*_2_)) for both exposed and unexposed subjects. When other parameters were fixed, a spike goes higher with the increase of the threshold. This allowed us to study how the volume of large *P* (*Y* = 1| *X*, *Z*_1_, *Z*_2_)) impacted model performance.

**Table 1:** Design of the simulation data

Scenario	Scenario				Models to generate simulation datasets^b^	Model misspe-cified?^c^
	Max P of exposed^a^	Beta of Z_2_	Link Function	% of exposed at Max P		
I-1	0.75	3	log	0	log (*P* (*Y*_1_ = 1| *X* = 0, *Z*_1_, *Z*_2_)) = −1.38 – *Z*_1_*–*3 * *Z*_2_	No
I-2	0.75	3	log	1.4	log (*P* (*Y*_2_ = 1| *X* = 0, *Z*_1_, *Z*_2_)) = − 1.23 – max (*Z*_1_ + 3 * *Z*_2_, 0.15)	Yes
I-3	0.75	3	log	2.8	log (*P* (*Y*_3_ = 1| *X* = 0, *Z*_1_, *Z*_2_)) = − 1.08 – max (*Z*_1_ + 3 * *Z*_2_, 0.30)	Yes
I-4	0.75	3	log	5.8	log (*P* (*Y*_4_ = 1| *X* = 0, *Z*_1_, *Z*_2_)) = − 0.78 – max (*Z*_1_ + 3 * *Z*_2_, 0.60)	Yes
II-1	0.85	3	log	0	log (*P* (*Y*_1_ = 1| *X* = 0, *Z*_1_, *Z*_2_)) = − 1.26 – *Z*_1_*–*3 * *Z*_2_	No
II-2	0.85	3	log	1.4	log (*P* (*Y*_2_ = 1| *X* = 0, *Z*_1_, *Z*_2_)) = − 1.11 – max (*Z*_1_ + 3 * *Z*_2_, 0.15)	Yes
II-3	0.85	3	log	2.8	log (*P* (*Y*_3_ = 1| *X* = 0, *Z*_1_, *Z*_2_)) = − 0.96 – max (*Z*_1_ + 3 * *Z*_2_, 0.30)	Yes
II-4	0.85	3	log	5.8	log (*P* (*Y*_4_ = 1| *X* = 0, *Z*_1_, *Z*_2_)) = − 0.66 – max (*Z*_1_ + 3 * *Z*_2_, 0.60)	Yes
III-1	0.95	3	log	0	log (*P* (*Y*_1_ = 1| *X* = 0, *Z*_1_, *Z*_2_)) = − 1.15 – *Z*_1_*–*3 * *Z*_2_	No
III-2	0.95	3	log	1.4	log (*P* (*Y*_2_ = 1| *X* = 0, *Z*_1_, *Z*_2_)) = − 1.00 – max (*Z*_1_ + 3 * *Z*_2_, 0.15)	Yes
III-3	0.95	3	log	2.8	log (*P* (*Y*_3_ = 1| *X* = 0, *Z*_1_, *Z*_2_)) = − 0.85 – max (*Z*_1_ + 3 * *Z*_2_, 0.30)	Yes
III-4	0.95	3	log	5.8	log (*P* (*Y*_4_ = 1| *X* = 0, *Z*_1_, *Z*_2_)) = − 0.55 – max (*Z*_1_ + 3 * *Z*_2_, 0.60)	Yes
IV-1	0.95	2	log	0	log (*P* (*Y*_1_ = 1| *X* = 0, *Z*_1_, *Z*_2_)) = − 1.15 – *Z*_1_*–*2 * *Z*_2_	No
IV-2	0.95	2	log	1.4	log (*P* (*Y*_2_ = 1| *X* = 0, *Z*_1_, *Z*_2_)) = − 1.05 – max (*Z*_1_ + 2 * *Z*_2_, 0.10)	Yes
IV-3	0.95	2	log	2.8	log (*P* (*Y*_3_ = 1| *X* = 0, *Z*_1_, *Z*_2_)) = − 0.95 – max (*Z*_1_ + 2 * *Z*_2_, 0.20)	Yes
IV-4	0.95	2	log	5.8	log (P (Y4 = 1| X = 0, Z1, Z2)) = − 0.75 – max (Z1 + 2 * Z2, 0.40)	Yes
V-1	0.95	4	log	0	log (*P* (*Y*_1_ = 1| *X* = 0, *Z*_1_, *Z*_2_)) = − 1.15 – *Z*_1_*–*4 * *Z*_2_	No
V-2	0.95	4	log	1.4	log (*P* (*Y*_2_ = 1| *X* = 0, *Z*_1_, *Z*_2_)) = − 0.95 – max (*Z*_1_ + 4 * *Z*_2_, 0.20)	Yes
V-3	0.95	4	log	2.8	log (*P* (*Y*_3_ = 1| *X* = 0, *Z*_1_, *Z*_2_)) = − 0.75 – max (*Z*_1_ + 4 * *Z*_2_, 0.40)	Yes
V-4	0.95	4	log	5.8	log (P (Y4 = 1| X = 0, Z1, Z2)) = − 0.35 – max (Z1 + 4 * Z2, 0.80)	Yes
VI-1	0.95	3	logit	0	logit (*P* (*Y*_1_ = 1| *X* = 0, *Z*_1_, *Z*_2_)) = − 0.76 – *Z*_1_*–*3 * *Z*_2_	Yes
VI-2	0.95	3	logit	1.4	logit (*P* (*Y*_2_ = 1| *X* = 0, *Z*_1_, *Z*_2_)) = − 0.61 – max (*Z*_1_ + 3 * *Z*_2_, 0.15)	Yes
VI-3	0.95	3	logit	2.8	logit (*P* (*Y*_3_ = 1| *X* = 0, *Z*_1_, *Z*_2_)) = − 0.46 – max (*Z*_1_ + 3 * *Z*_2_, 0.30)	Yes
VI-4	0.95	3	logit	5.8	logit (*P* (*Y*_4_ = 1| *X* = 0, *Z*_1_, *Z*_2_)) = − 0.16 – max (*Z*_1_ + 3 * *Z*_2_, 0.60)	Yes
VII-1	0.95	3	probit	0	probit (*P* (*Y*_1_ = 1| *X* = 0, *Z*_1_, *Z*_2_)) = − 0.48 – *Z*_1_*–*3 * *Z*_2_	Yes
VII-2	0.95	3	probit	1.4	probit (*P* (*Y*_2_ = 1| *X* = 0, *Z*_1_, *Z*_2_)) = − 0.33 – max (*Z*_1_ + 3 * *Z*_2_, 0.15)	Yes
VII-3	0.95	3	probit	2.8	probit (*P* (*Y*_3_ = 1| *X* = 0, *Z*_1_, *Z*_2_)) = − 0.18 – max (*Z*_1_ + 3 * *Z*_2_, 0.30)	Yes
VII-4	0.95	3	probit	5.8	probit (*P* (*Y*_4_ = 1| *X* = 0, *Z*_1_, *Z*_2_)) = − 0.12 – max (*Z*_1_ + 3 * *Z*_2_, 0.60)	Yes

^a^Maximum *P* (*Y*_*k*_ = 1| *X* = 1, *Z*_1_, *Z*_2_)

^b^Models to generate *Y*_*k*_ for unexposed subjects. For exposed subjects, *P* (*Y*_k_ = 1| *X* = 1, *Z*_1_, *Z*_2_) =3*P (*Y*_k_ = 1| *X* = 0, *Z*_1_, *Z*_2_). k = 1, 2, 3, and 4

^c^Model was defined as misspecified when the link function was not ‘log’ or the % of exposed at maximum *P* (*Y*_k_ = 1| *X* = 0, *Z*_1_, *Z*_2_) was greater than 0

#### Scenarios to study the impact of maximum P(*Y* = 1)

First, scenarios I-1, II-1, and III-1 were created using the log link function. The maximum values of *P* (*Y*_*1*_ = 1| *X* = 1, *Z*_1_, *Z*_2_)) were set to 0.75, 0.85 and 0.95, respectively, to study the impact of maximum *P* (*Y*_*1*_ = 1| *X* = 1, *Z*_1_, *Z*_2_)) (Table 1). The selected thresholds were set at 0.15, 0.30, and 0.60 for *Y*_2_, *Y*_3,_ and *Y*_4_, respectively. The percentages of exposed subjects who were at the maximum values set above (0.75, 0.85, and 0.95) were 1.4, 2.8, and 5.8%. These values were derived by simulation and represented the various levels of alteration of the linear predictors.

The intercepts were manually calculated to satisfy *P* (*Y*_*k*_ = 1 | *X* = 0, *Z*_1_, *Z*_2_) ≤ 0.75/3, 0.85/3 and 0.95/3, respectively. For example, for the equation log (*P* (*Y*_1_ = 1| *X* = 0, *Z*_1_, *Z*_2_)) = α – *Z*_1_*–*3 * *Z*_2_ in scenario I-1, α = log(0.75/3) = − 1.38 since the logarithm is an increasing function and hence the maximum of *P* (*Y*_1_ = 1| *X* = 0, *Z*_1_, *Z*_2_) is achieved when *Z*_1_ *=* 0 and *Z*_2_ = 0. For the same reason, for the equation log (*P* (*Y*_2_ = 1| *X* = 0, *Z*_1_, *Z*_2_)) = α – *Z*_1_ – max (*Z*_1_ + 3 * *Z*_2_, 0.15) in scenario I-2, α = log(0.75/3) + 0.15 = − 1.23. The 12 scenarios designed to study the impact of large *P*(*Y = 1*) were listed in the first section of Table 1 (in the first 12 rows).

#### Scenarios to study the impact of coefficient of *Z*_2_

To study the impact of the entire distribution of *P(Y = 1)* when large *P(Y = 1)* existed, eight more scenarios were produced, with the beta coefficient of *Z*_2_ being 2 and 4 (shown in the middle section of Table 1), to join the four scenarios for which the beta coefficient of *Z*_2_ was set to 3 (i.e. III-1, III-2, III-3, and III-4). The distribution of *P(Y = 1)* was shifted towards zero as the beta coefficient of *Z*_2_ increased. Thus, these scenarios allowed us to study the impact of the outcome distribution, or the average *P(Y = 1)*.

The intercepts and the thresholds were generated using the same approach as described in the previous section. Because *Z*_2_ follows the uniform distribution, the thresholds increase proportionally with the beta coefficients. For example, the threshold to make 1.4% of exposed subjects reached the maximum *P* (*Y*_*2*_ = 1| *X* = 1, *Z*_1_, *Z*_2_) = 0.1 when the beta of *Z*_2_ was 2 and increased to 0.2 when the beta of *Z*_2_ was 4.

#### Scenarios to study the impact of misspecified link functions

The link function was altered from log to logit and probit in scenarios VI and VII to assess the model performance when the link functions were misspecified; refer to the last section of Table 1. For scenarios VI-2, VI-3, VI-4, VII-2, VII-3, and VII-4, not only were the link functions misspecified, but also the responses depended on covariates with truncated probabilities.

#### Scenarios with a weaker association between exposure and outcome (RR = 2)

To understand the impact of misspecified link functions and truncation when RR is different from 3.0, we also generated scenarios with parameters identical to those in III-1, III-2, III-3, III-4, VII-1, VII-2, VII-3 and VII-4, except that this time RR = 2.0 instead of 3.0.

## Results

### Relative bias

**Fig. 1:**
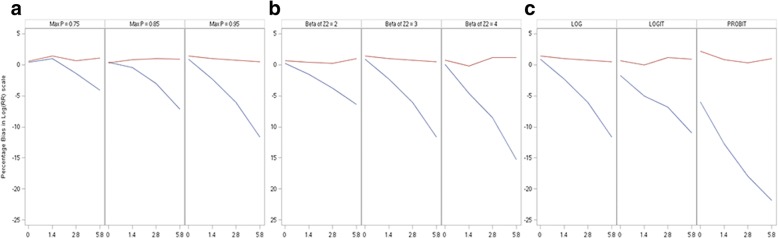
Percentage bias in log(RR) scale. **a** From left to right: increasing intercept(scenario I→II →III); **b** From left to right: increasing coefficient of β_2_ (scenario IV→III→V); **c** From left to right: change of link function (scenarios III, VI and VII). Red lines: Robust Poison; Blue lines: Log-binomial

The relative biases of the estimated *RR* in log scale from the two models in each of the 28 scenarios when *n* = 1000 are shown in Table 2 and Fig. 1.

**Table 2:**
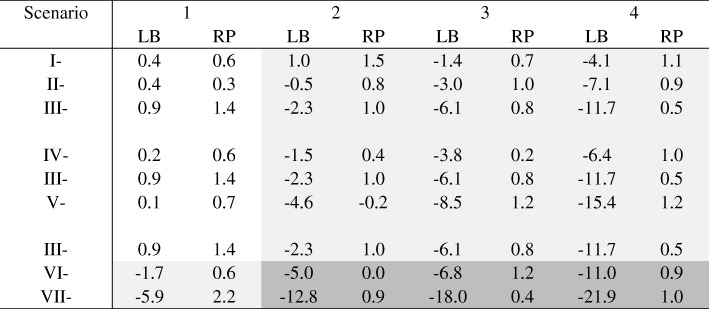
Relative bias (%) in log scale with and without model misspecification (*n* = 1000)

Unshaded: Models were correctly specified

Light shaded: Misspecified linear predictors or misspecified link function

Dark shaded: Misspecified linear predictors and misspecified link function

LB: log-binomial, RP: robust Poisson

Change of scenarios: Increasing intercept: I → II → III; Increasing coefficient of β_2_: IV → III → V; Change of link function: III (log), VI (logit), VII (probit)

As expected, both models accurately estimated *β*_1_ or log (*RR*) when they were correctly specified, regardless of the value of the maximum *P*. When the models were misspecified (as shown in the shaded areas in Table 2), the relative biases of the robust Poisson models were negligible, while those of log-binomial models tended to negatively bias away from null. For the log-binomial models, the magnitude of biases increased in all scenarios when the level of misspecification, measured by the percentage of exposed subjects at maximum *P*, increased.

Large *P(Y = 1)* was associated with an increased level of bias when the percentage of exposed subjects having the max *P* (*Y*_*k*_ = 1| *X* = 1, *Z*_1_, *Z*_2_) was fixed; refer to the first three rows of Table 2 and Fig. 1, Panel A. For example, when the percentage of exposed subjects whose maximum *P* (*Y*_*k*_ = 1| *X* = 1, *Z*_1_, *Z*_2_) was fixed at 5.8, an increase of maximum *P* (*Y*_*k*_ = 1| *X* = 1, *Z*_1_, *Z*_2_) from 0.75 (scenario I-4) to 0.95 (scenario III-4) resulted a change in relative bias from − 4.1% to − 11.7%.

The impact of average *P(Y = 1)* was displayed from the 4th to the 6th rows of Table 2 and in Panel B of Fig. 1. When the percentage of exposed subjects having the max *P* (*Y*_*k*_ = 1| *X* = 1, *Z*_1_, *Z*_2_) was fixed, the log-binomial models were more vulnerable (larger absolute value of relative biases) when the beta coefficient of *Z*_2_ increased (i.e. average *P* (*Y* = 1| *X* = 1, *Z*_1_, *Z*_2_) decreased). For example, when the percentage of exposed subjects whose maximum *P* (*Y*_*k*_ = 1| *X* = 1, *Z*_1_, *Z*_2_) = 2.8, the relative bias changed from − 3.8% to − 8.5% when the beta coefficient increased from 2 to 4. This indicates that the value of the average *P(Y = 1)* impacts the performance of the log-binomial models. When there were enough large *P(Y = 1)*, a low average *P(Y = 1)* away from large *P(Y = 1)* was associated with a large relative bias.

When the underlying distribution of data was logit, misspecifying the link function as ‘log’ did not significantly influence relative biases. However, when the underlying distribution of data was probit, the bias (− 5.9%) was noticeable even when the linear predictors were properly specified (i.e. percentage of exposed subjects having the max *P* (*Y*_*k*_ = 1| *X* = 1, *Z*_1_, *Z*_2_) was zero). Refer to the last three rows of Table 2 and Panel C of Fig. 1. Misspecifying the link function from log to probit in the presence of misspecified linear predictors had a serious consequence. The relative bias was almost − 18% when the percentage of exposed subjects at the maximum *P* was only 2.8.

### Standard error

In all simulation scenarios, the SE of the two models were comparable (Table 3). At the 2nd decimal point, the SE derived from the log-binomial models were either the same or slightly smaller compared to those of robust Poisson models. The largest difference, 0.03 (=0.23–0.20), occurred when the data distribution was probit, maximum *P* (*Y* = 1| *X* = 1, *Z*_1_, *Z*_2_) was 0.95, and the beta of Z_2_ was 3 (scenario VII-1). When the models correctly specified (in the unshaded area of Table 3), the SE of the two models were the same except for one scenario (V-1) in which the SE from log-binomial models was smaller by 0.01. The SE of the log-binomial models were always smaller compared to those of robust Poisson models at the 3rd decimal place, even if the models were correctly specified (data not shown).

**Table 3:**
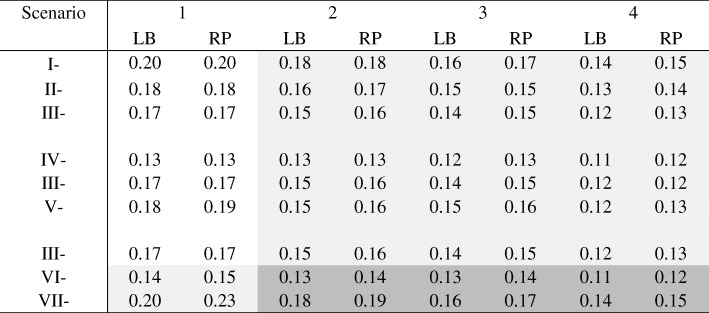
Standard error in log scale with and without model misspecification (n = 1000)

Unshaded: Models were correctly specified

Light shaded: Misspecified linear predictors or misspecified link function

Dark shaded: Misspecified linear predictors and misspecified link function

LB: log-binomial, RP: robust Poisson

Change of scenarios: Increasing intercept: I → II → III; Increasing coefficient of β_2_: IV → III → V; Change of link function: III (log), VI (logit), VII (probit)

### Mean square error

As expected, when the models were correctly specified (in the unshaded area in Table 4), log-binomial models yielded the same or marginally smaller MSE compared to robust Poisson models. When the underlying distribution of data had a log or logit link (scenarios I-VI) and the percentage of exposed subjects having the max *P* (*Y*_*k*_ = 1| *X* = 1, *Z*_1_, *Z*_2_) was 1.4% or 2.8% (in the columns labeled as ‘2’ and ‘3’ in Table 4), the MSE of the two models were still comparable, except for one scenario. The exception occurred in scenario V-3, where the superiority of the robust Poisson models was quite noticeable; the difference in MSE between the two models was 0.05. Recall that the average *P(Y = 1)* for V-3 was smallest among all three scenarios (III-3, IV-3 and V-3) and followed by that of III-3. Thus, it is not surprising to observe the difference between the two models becoming larger from IV-3 to III-3, and to V-3. For scenarios III-4 to VII-4, the robust Poisson models consistently outperformed the log-binomial models. For scenarios of VII (in which the underlying data were generated using a probit link), the MSE of log-binomial models, compared to those of robust Poisson models, were slightly smaller when the linear predictors were properly specified (such as in scenario VII-1) and significantly larger when the linear predictors were improperly specified, even when level of misspecification of the linear predictors was small. For example, when the percentage of exposed subjects at the 0.95 (max *P*) was only 1.4, the MSE were 0.051 and 0.038, from the log-binomial model and the robust Poisson model, respectively.

**Table 4:**
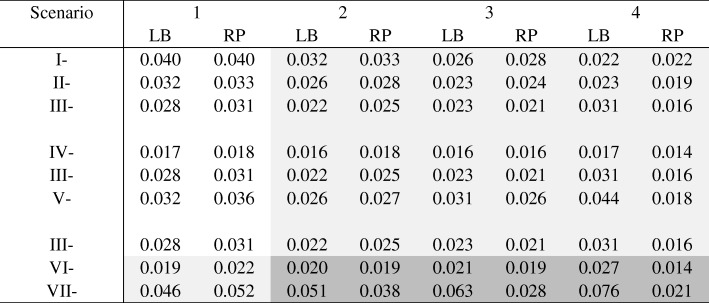
Mean square error (MSE) in log scale with and without model misspecification (n = 1000)

Unshaded: Models were correctly specified

Light shaded: Misspecified linear predictors or misspecified link function

Dark shaded: Misspecified linear predictors and misspecified link function

LB: log-binomial, RP: robust Poisson

Change of scenarios: Increasing intercept: I → II → III; Increasing coefficient of β_2_: IV → III → V; Change of link function: III (log), VI (logit), VII (probit)

### Distribution of P(Y = 1)

**Fig. 2:**
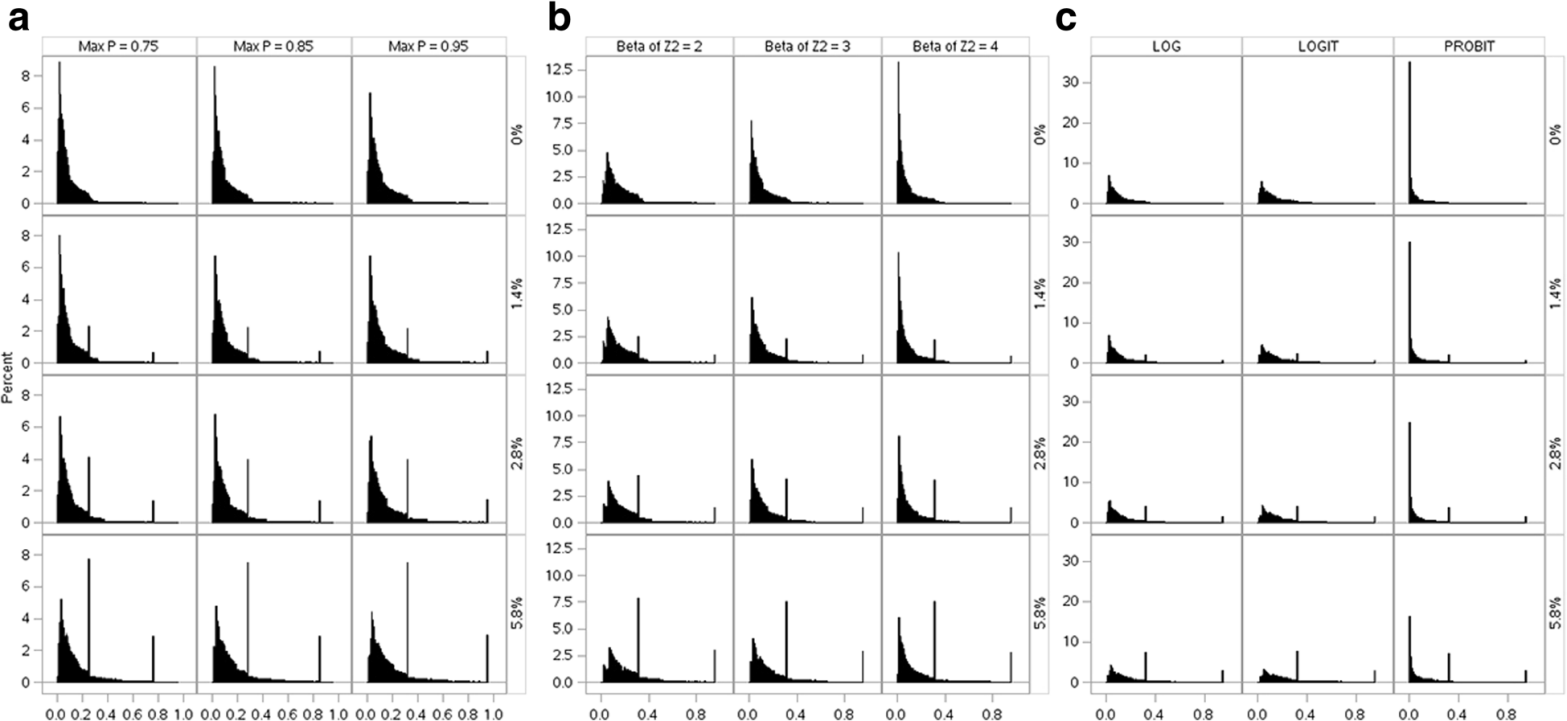
Distribution of P(Y = 1). Y-axis: Percent; X-axis: P(Y = 1). **a** From left to right: increasing intercept (scenario I→II→III); **b** From left of right: increasing coefficient of β_2_ (scenarion IV→III→V); **c** From left to right: change of link function (scenarios III, VI and VII)

The distributions of *P(Y = 1)* for all simulated data (one million data points for each scenario) are shown in Fig. 2. Panel A shows the distribution of *P(Y = 1)* with varying maximum *P(Y = 1)*. When the link function and beta of *Z*_2_ were fixed at ‘log’ and 3, respectively, an increase of max *P(Y = 1)* from 0.75 (scenario I) to 0.95 (scenario III) stretched the spikes to the right (Fig. 2 Panel A).

Panel B shows the distribution with varying beta of *Z*_2_. When the beta of *Z*_2_ increased from 2 (scenario VI) to 4 (scenario V) while the max *P(Y = 1)* was fixed at 0.95 and the link function was set as ‘log,’ the distribution of *P(Y = 1)* became taller, thinner, and shifted towards zero. This provides the evidence that attributes the increase of biases to the downward shift of the distribution of *P(Y = 1)* (i.e. decrease in the prevalence of the outcome variable *Y*).

Panel C shows the distribution with varying link function. Spikes (i.e. vertical thin lines in all figures except those labeled with “0% at the max P”) for both exposed and unexposed subjects increased in all scenarios when the percentage of exposed subjects at the max *P(Y = 1)* increased from 1.4, 2.8 to 5.8%, indicating a higher level of violation of the linearity assumption. The distributions of *P(Y = 1)* for data with log and logit link functions were similar; however, the distributions of *P(Y = 1)* for data with probit link function distinguished themselves significantly from those of log or logit. This observation explains the larger biases seen in Fig. 1 Panel C, when the underlying distribution of data were probit, compared to those of log and logit distributions, even when the predictors were perfectly linear.

### Results of moderate sample size (*n* = 500)

When the simulation was conducted based on samples of moderate size (n = 500), the same pattern was observed in terms of relative biases and SE (Additional file 6, Tables AF6.1-AF6.2). As expected, the SE based on the samples of moderate size was larger compared to those derived from samples with *n* = 1000. The same pattern was also observed for MSE (Additional file 6, Table AF6.3); however, the differences between the two models were not as substantial as seen in the samples of sizes 1000.

### Results with a weaker association between exposure and outcome (RR = 2)

When the simulation was conducted based on simulation datasets with RR = 2, the relative biases were similar to those of the corresponding scenarios with RR = 3 (data not shown). The standard errors derived from the simulation datasets with RR = 2 were 12–36% smaller compared to those of the corresponding scenarios with RR = 3; however, the pattern remained the same. That is, the standard errors of the two models were comparable with those from the robust Poisson models being only slightly larger than those from the log-binomial models. The MSE yielded from the simulation datasets with RR = 2 were 16–47% smaller compared to those of the matching datasets with RR = 3. Nevertheless, similar to the patterns observed for RR = 3, the robust Poisson had lower MSE compared to the log-binomial models, and the differences were more dramatic when a probit link was used (versus a log link) and when the data had a higher percentage of truncation.

## Discussion

In this study, the statistical performances of the two most popular model-based approaches used to estimate *RR* for common binary outcomes were examined when the link function was misspecified or when the probability of the response variables was truncated at the right tail. Our findings suggest that point estimates from log-binomial models were biased when the link function was misspecified or when the probability distribution of the response variable was truncated for even a small proportion of observations. For log-binomial models, the percentage of truncated observations was positively associated with the presence of the bias. The bias was more significant if these observations came from a population in which the response rate was lower, given the other parameters being examined were fixed.

For MLE based methods, misspecification can cause inconsistent estimators of parameters [20]. Lumley et al. (2006) pointed out that compared to robust Poisson and other non-MLE based models, log-binomial models (MLE based) have very large weights when p (referred by authors as μ) is large ([26] Fig. 1). The same authors also pointed out that for log-binomial models, “a single point with μ close to 1 can have arbitrarily large influence despite having bounded covariate values”. Our observation was consistent with that of Lumley et al. We demonstrated that when the percentage of observations with large P increased, the magnitude of bias also increased. This may explain the less optimal performance of the model applied to data generated using a probit link compared to that of log or logit link when other parameters were fixed (Panel C of Fig. 2). It is well known that log-binomial models may fail to convergence or generate incorrect estimates when the covariate values in the data are not bounded by 1 [3, 5, 8]. However, we believe that this is a different issue than what we have focused on, which has been the impact of large Ps. In Scenario VII-1, truncation was not applied and none of the observations had predicted probabilities > 1, yet the point estimate was still biased.

On the other hand, the point estimates from the robust Poisson models were nearly unbiased in all the scenarios examined, including when they were applied to the data that were generated using a probit link, which yielded quite different probability distributions compared to those from a log link, and/or when the distribution of 5.8% of the exposed subjects were altered. In Chen et al. [27], both the MLE generated by log-binomial models and the quasi-likelihood estimators produced by robust Poisson models deteriorated when outliers were introduced [27]. However, in the current study, the biases in point estimates based on robust Poisson models were negligible, even when both link functions and predictors were incorrectly specified. This interesting contrast can be explained by a major difference in the design of the two studies. In the previous study [27], the association between the exposure and the outcome was weakened when the “outliers” were introduced, and thus the negative biases were observed for the robust Poisson models. Nevertheless, in the current study, the true *RR* was maintained at 3.0, (or 2.0 for some scenarios), even when the link function was misspecified and/or when the probabilities were truncated. Our simulations demonstrated that for robust Poisson regression, the misspecification of the link function did not hinder its ability to find the true *RR*. This is likely due to the fact that the quasi-likelihood method enables regression coefficient estimation without fully specifying the distribution of the observed data. We examined exposure-outcome associations with RR 3.0 and 2.0. The magnitude of the observed bias in our simulation results did not change much when the association was reduced from 3.0 to 2.0; however, it is conceivable that the bias could be reduced in scenarios when the association is smaller than 2.0.

Model misspecification does not always yield differences in point estimates between the two models. In fact, in a previous examination (Additional file 3), we found when an important explanatory variable was omitted, a higher order term of non-linear explanatory variable was ignored, or an interaction term was overlooked, the two models produced comparable results regardless of the outcome rate, risk ratio or the strength of association between the exposure and the confounder or between the outcome and the confounder. Only in the scenario where an interaction term was ignored did the models yield large biases. This highlights the relative importance of observations with large weights, since in the previous examination, the number of observations with large probabilities of having the response was small.

Although we did not evaluate data based on other link functions that are also suitable for modeling binary outcomes (e.g. complementary log-log or log-log), it is expected that the results would have similar patterns. A truncated distribution appears in many real-life datasets where the collection of data is limited to those that are above or under a threshold. For example, a typical scale used in clinics or hospitals can measure height up to 200 cm and weight up to 250 kg. Subjects exceeding these values would be truncated to these limits. In the simulated datasets, the distributions of approximately 1.4, 2.8, and 5.8% of the exposed subjects were truncated in that they no longer followed the distribution specified by the link function through a combination of linear predictors. The truncation rates (1.4, 2.8, and 5.8%) for the exposed subjects were plausible values that can be related to real-life applications.

In contrast to Chen et al. [27], in which no differences were found at the second decimal point when the data were not contaminated with outliers, we found small differences in the variances at the second decimal point between the log-binomial and robust Poisson models under some of the scenarios for both samples (*n* = 1000 and *n* = 500) when the models were correctly specified. This finding is consistent with that of Petersen and Deddens [11], which was based on a sample with 100 observations and a single independent variable with a uniform distribution.

Kauermann and Carroll [28] showed that variances of sandwich estimators were generally less efficient than variance estimates derived from parametric models. This weakness impacts the coverage probability, the probability that a confidence interval covers the true *RR*, and thus the ability to reject a null when the alternative is true. Hence, log-binomial models are preferred over the robust Poisson models when the log-binomial models are correctly specified.

The COPY method was reported to have convergence issue when there are continuous covariates in the model [11]. However, convergence was barely an issue in this study as it converged completely (i.e. 1000 out of 1000 simulations) in 23 out of 28 scenarios when the sample size was 1000, and 21 out of 28 scenarios when the sample size was 500. In the 12 scenarios (five for sample size 1000 and seven for sample size 500) for which the COPY method did not completely converge in all 1000 simulations, there was only one out of 1000 simulations that failed to converge for each scenario. The number of virtual copies used in the study, 10,000, was reported to be accurate to three decimal places [25].

Misspecification tests were developed [29, 30] and proven to be able to maintain reasonable size across various settings in simulation when they were applied to logistic and beta-binomial regression models [30]. However, the power to detect the types of alternatives commonly observed in practice (e.g. alternative link functions) was low [30]. Blizzard and Hosmer [10] assessed model-fit of log-binomial models by applying the Hosmer-Lemeshow test (a commonly used goodness-of-fit test for logistic regression models), the Pearson chi-square test, and the unweighted sum of squares test, finding that all three tests exhibited acceptable Type I errors yet low-to-moderate power. Due to the lack of powerful diagnostic tools to detect any forms of model misspecification, the robust Poisson model may be considered a good choice because of its ability to produce unbiased risk ratios. Efforts to establish efficient and robust parameter estimators are ongoing. A recent publication summarized issues with the current approaches within the GLM family to estimate relative risks and risk differences, and provided a possible alternative to estimate relative risks and risk differences using a non-GLM approach [31]. The authors proposed to model relative risks as functions of baseline covariates. Validation of this approach is needed to determine its applicability to studies such as those presented here.

## Conclusions

Given the vulnerability of log-binomial models when they are misspecified, a robust Poisson model should be considered the preferred choice for estimating risk ratios. This is especially the case when the prevalence of the outcome is low and the model contains continuous covariates. If the result of a robust Poisson model approaches borderline significance, consider performing a log-binomial regression as well, as the increased efficiency of the log-binomial model may increase the probability of detecting the effect with a given significance level. If the point estimates of the two models are inconsistent and the log-binomial model is preferred, categorize continuous variables and re-fit the model. If the data contain truncated values, examine the distribution of the data carefully and consider converting them into categorical variables if such a conversion is clinically meaningful. The robust Poisson model does not work well for samples that are very small because the sample-based sandwich estimators tend to underestimate the true standard errors [32].

In summary, we found evidence to favor the robust Poisson model under various scenarios when models were misspecified. Future studies to develop model misspecification and/or goodness-of-fit tests that are powerful and convenient to apply for log-binomial models are warranted.

## Additional files

Additional file 1: Popularity of log-binomial and robust Poisson regression models – A Medline search. (DOCX 18 kb)

Additional file 2: Comparison of robust Poisson and log-binomial models in estimating risk ratio (RR) of ≥ 7 SABA canisters dispensed in the past year. (DOCX 12 kb)

Additional file 3: Comparing robustness to model misspecification between robust Poisson and log-binomial models for estimating risk ratios: Initial simulation study. (DOCX 45 kb)

Additional file 4: Using Fish scoring (iteration) to estimate β. Proof of the iteration equation in Methods section. (DOCX 45 kb)

Additional file 5: SAS codes used to estimate β for each regression model. (DOCX 12 kb)

Additional file 6: Results on simulated data when *n* = 500. (DOCX 18 kb)

## Abbreviations

ACT: Asthma control test
FeNO: Fractional exhaled nitric oxide
FEV: Forced expiratory volume
GEE: Generalized estimation equation
GLM: Generalized linear models
ICS: Inhaled corticosteroids
IRLS: Iterative reweighted least squares
MLE: Maximum likelihood estimator
MSE: Mean square error
QL: Quasi-likelihood
RR: Risk ratio
SABA: Short-acting beta-agonist
SE: Standard error
