# Supplementary material for: Comparing performance between log-binomial and robust Poisson regression models for estimating risk ratios under model misspecification
Source: BMC Med Res Methodol. 2018 Jun 22;18:63. doi: 10.1186/s12874-018-0519-5 (PMC6013902; doi:10.1186/s12874-018-0519-5)
Supplement: Supplementary file 1 — Popularity of log-binomial and robust Poisson regression models – A Medline search. (DOCX 18 kb) [file 12874_2018_519_MOESM1_ESM.docx]

**Additional file 1. Popularity of Log-binomial and Robust Poisson Regression Models – A Medline Search**

A Medline search of articles published between 2005 and 2014 was conducted to understand the popularity of the log-binomial and the robust Poisson models. The search phrases were “log binomial regression” for log-binomial models, and (“modified Poisson regression”) OR (“robust Poisson regression”) for robust Poisson models. This resulted in the same results of the Boolean search based on the following search terms: “log” AND “binomial” AND “regression” for log-binomial models, and (“modified” AND “Poisson” AND “regression”) OR (“robust” AND “Poisson” AND “regression”) for robust Poisson models. The Medline search engine is designed to find papers with the matching keywords defined by the authors, even if the keywords do not appear consecutively or do not appear at all in the abstracts or in the main body of the articles. The search was limited to “human” studies.

A total of 461 and 564 articles were initially identified for log-binomial regression models and for modified or robust Poisson regression models, respectively. A manual review was performed to exclude studies that utilized statistical methods that are not related to log-binomial or robust Poisson models (e.g. negative binomial regression models or zero-inflated Poisson regression models). Studies that applied log-binomial or robust Poisson models for purposes other than estimating risk ratios were also excluded (e.g. Poisson regression models to estimate counts, rates, rate ratios or differences). Moreover, extensions of log-binomial models or robust Poisson models to handle repeated or clustered measures were removed because the use is beyond the scope of the current study. The reasons for exclusion and the number of articles being excluded are listed in Table AF1.1.

Table AF1.1. Medline search exclusion criteria and number of articles excluded

| “log-binomial regression”   - Negative binomial regression models - Statistical methods research - Log-binomial models with generalized estimating equation (GEE) - Logistic regression models - Log-binomial with a hierarchical approach - Other (i.e. identified because of the appearance of key words) | 36  24  23  9  5  4 |
| --- | --- |
| “robust Poisson regression” or “modified Poisson regression”   - Poisson regression models to estimate counts, rates, rate ratios or differences - Poisson two stage, multi-level, hierarchical, random effect or mixed effect models - Robust or modified Poisson models with generalized estimating equation (GEE) - Statistical methods research on robust or modified Poisson models - Statistical methods research on other types of Poisson models (excludes ZIP) - Zero-inflated Poisson (ZIP) - Regression with distributions other than Poisson (e.g. Logistic) - Poisson regression to estimate risk ratio. Unclear how the variance was estimated - Other with the key words including 1 review paper and 2 papers on meta-analysis | 73  27  16  12  15  8  9  8  20 |

Table AF1.2 displays the number of articles that utilized log-binomial regression models and robust Poisson regression models per year between 2005 and 2014 after the exclusions listed in Table AF1.1. The volumes increased significantly during the 11 years. Three studies performed both and thus they were counted in both columns in Table AF1.2. Four out of 360 (376) studies in which log-binomial (robust Poisson) models were applied were clinical trials.

Table AF1.2. Number of publications in 2005-2014 using log-binomial and/or

robust (modified) Poisson regression models

| Year | Log-binomial models^1^ | Robust (modified) Poisson models^2^ |
| --- | --- | --- |
|  |  |  |
| 2005 | 8 | 2 |
| 2006 | 10 | 9 |
| 2007 | 16 | 8 |
| 2008 | 20 | 21 |
| 2009 | 29 | 20 |
| 2010 | 39 | 27 |
| 2011 | 38 | 46 |
| 2012 | 58 | 70 |
| 2013 | 67 | 88 |
| 2014 | 75 | 85 |
| Total | 360 | 376 |

1. Four out of 360 were clinical trials and the rest were observational studies.
2. Four out of 376 were clinical trials and the rest were observational studies
