# Supplementary material for: Comparing performance between log-binomial and robust Poisson regression models for estimating risk ratios under model misspecification
Source: BMC Med Res Methodol. 2018 Jun 22;18:63. doi: 10.1186/s12874-018-0519-5 (PMC6013902; doi:10.1186/s12874-018-0519-5)
Supplement: Supplementary file 2 — Comparison of robust Poisson and log-binomial models in estimating risk ratio (RR) of ≥ 7 SABA canisters dispensed in the past year. (DOCX 12 kb) [file 12874_2018_519_MOESM2_ESM.docx]

**Additional file 2. Comparison of robust Poisson and log-binomial models in estimating risk ratio (RR) of >7 SABA canisters dispensed in the past year**

| FeNO | Robust Poisson RR (95% CI) | | Log-Binomial RR (95% CI) | |
| --- | --- | --- | --- | --- |
|  | Unadjusted | Adjusted | Unadjusted | Adjusted |
| Reference: 1^st^ quartile (7-19 ppb) |  |  |  |  |
| 2^nd^ quartile (20-28 ppb) | 1.94 (0.95-3.99) | 2.05 (1.03-4.05)* | 1.94 (0.97-4.17) | 1.67 (0.83-3.57) |
| 3^rd^ quartile (29-47 ppb) | 1.56 (0.73-3.31) | 1.40 (0.68-2.85) | 1.56 (0.74-3.43) | 1.23 (0.58-2.72) |
| 4^th^ quartile (48-215 ppb) | 3.37 (1.77-6.42)* | 2.51 (1.31-4.79)* | 3.37 (1.84-6.84)* | 2.11 (1.10-4.45)* |

Variables adjusted in the models were age, gender, race/ethnicity, number of aeroallergen sensitivities, FEV_1_% predicted

(<80% vs. ≥80%), asthma control test score (<16, 16-19, >19) and clinical center.

***** Statistically significant at the 95% level.
