# Supplementary material for: Comparing performance between log-binomial and robust Poisson regression models for estimating risk ratios under model misspecification
Source: BMC Med Res Methodol. 2018 Jun 22;18:63. doi: 10.1186/s12874-018-0519-5 (PMC6013902; doi:10.1186/s12874-018-0519-5)
Supplement: Supplementary file 3 — Comparing robustness to model misspecification between robust Poisson and log-binomial models for estimating risk ratios: Initial simulation study. (DOCX 45 kb) [file 12874_2018_519_MOESM3_ESM.docx]

**Additional file 3. Comparing Robustness to Model Misspecification between Robust Poisson and Log-Binomial Models for Estimating Risk Ratios: Initial Simulation Study**

**Simulation Methods**

Simulation was conducted to examine the performance of the log-binomial and the robust Poisson regression models for estimating risk ratios (RR). The two models were compared when the following types of misclassification occurred:

1. Ignoring one of the two confounders
2. Ignoring the quadratic term when the confounder is quadratically associated with the outcome
3. Ignoring the interaction between the exposure and the confounder

**Notation**

x is a binary exposure that is fixed at 50% in all the scenarios. y is a binary outcome. z, z_1_, z_2_ are continuous confounders with beta (6,2) distribution.

Data-generating process

Six scenarios were generated based on the combination of the following characteristics:

(1) RR = 1.0, 1.5, 2.0

(2) y = 10%, 25%

We first generated a random variable z (or two random variables z_1_ and z_2_ in sections a) below) using beta (6,2) distribution. Then we generated the exposure status for each of the n subjects based on the subject-specific probability of exposure using logistic regression. For each of the n subjects, we randomly generated an outcome conditional on the exposure status and covariates using Log-Binomial regression. The regression coefficients (α_1_, β_1_, β_2_, β_3_) were defined as log(2) or log(4) to indicate moderate and strong association, respectively, between x (or y) and z (or z_1_, z_2_). For example, in scenario b2 below, z is moderately associated with x but strongly associated with y. The value of β_1_ in all the models denotes the effect due to exposure on the log-relative risk scale. The values of α_0_ and β_0_ were searched iteratively among all the possible values to guarantee that half of the subjects exposed (x=50%) and the outcome rate was 10% or 25%. The beta distribution was chosen to generate Z because it is supported on a closed range [0,1] and therefore finding a finite β_0_ is possible during the step in which we generated the outcome variable.

1. Generating the scenarios with two confounders.

logit(p_i,x_)=α_0_+α_1_z_1_+α_1_z_2_, log(p_i,y_)=β_0_+β_1_x+β_2_z_1_+β_2_z_2_, z_1_ and z_2_ are confounders

Sub-scenarios:

a1: α_1_=log(2), β_2_=log(2), z_1_ and z_2_ are moderately associated with both x and y

a2: α_1_=log(2), β_2_=log(4), z_1_ and z_2_ are moderately associated with x but strongly associated with y

a3: α_1_=log(4), β_2_=log(2), z_1_ and z_2_ are strongly associated with x but moderately associated with y

a4: α_1_=log(4), β_2_=log(4), z_1_ and z_2_ are strongly associated with both x and y

b) Generating the scenarios with a confounder that was quadratically associated with the outcome.

logit(p_i,x_)=α_0_+ α_1_z, log(p_i,y_)=β_0_+β_1_x+β_2_z+(0.5*β_2_)z^2^, z is a confounder

Sub-scenarios:

b1: α_1_=log(2), β_2_=log(2), z is moderately associated with both x and y

b2: α_1_=log(2), β_2_=log(4), z is moderately associated with x but strongly associated with y

b3: α_1_=log(4), β_2_=log(2), z is strongly associated with x but moderately associated with y

b4: α_1_=log(4), β_2_=log(4), z is strongly associated with both x and y

c) Generating the scenarios with an interaction term.

logit(p_i,x_)=α_0_+ α_1_z, log(p_i,y_)=β_0_+ β_1_x+ β_2_z +(0.7*β_2_)xz, z is not a confounder

Sub-scenarios:

c1: α_1_=log(1), β_2_=log(2), z is moderately associated with y but not associated with x

c2: α_1_=log(1), β_2_=log(4), z is strongly associated with y but not associated with x

**Estimation Methods**

The data were generated by using SAS/BASE and analyzed using SAS’s GENMOD, version 9.2 [1]. To address the non-convergence issue that often occurs for log-binomial models in many software packages, the COPY macro was used with C, the number of virtual copies, set to be 1 million [2,3].

For each of the 6 scenarios and 10 sub-scenarios (a total of 60 sub-scenarios), we randomly generated 2,000 datasets each with 500 subjects using the methods described in section C.2. The risk ratio was estimated using the log-binomial and the robust Poisson regression models when the models were misspecified in the following manner:

1. Ignoring the confounder z_2_. Only x and z_1_ were included in the model
2. Model the outcome using x and z, ignoring the term z^2^
3. Model the outcome using x and z, ignoring the interaction between x and z

The following performance measures were produced.

1. Percentage bias=$\left(\frac{\bar{\hat{r}}-r}{r}\right)*100$, where $\bar{\hat{r}}=\sum_{i=1}^{2000} r_{i}/2000$and r is the true effect of exposure in log scale.
2. Standardized bias=$\left(\frac{\bar{\hat{r}}-r}{SE(\hat{r})}\right)*100$, where $\bar{\hat{r}}=\sum_{i=1}^{2000} r_{i}/2000$, $SE(\hat{r})$is the empirical SE of the effect of exposure in log scale over 2,000 simulations and r is the true effect of exposure in log scale. A standardized bias of greater than 40% in either direction was reported to have noticeable impact on efficiency, coverage and error rates.
3. Mean square error (MSE)=$(\bar{\hat{r}}-r)^{2}+(SE(\hat{r}))^{2}$, where $\bar{\hat{r}}=\sum_{i=1}^{2000} r_{i}/2000$, $SE(\hat{r})$is the empirical SE of the effect of exposure in log scale over 2,000 simulations and r is the true effect of exposure in log scale.
4. 95% Coverage=Proportion of times the 95% Wald-type confidence interval (CI) $\hat{r}_{i}\pm Z_{0.975}SE(\hat{r}_{i})$include r for i=1,2,…,2000.

**Results**

In all scenarios, log-binomial and robust Poisson models yielded similar relative biases, standardized biases, 95% coverage probabilities and MSEs (Tables AF3.1-AF3.4). The biases were all positive, indicating that the estimated RRs tended to be higher than the true RRs (Table AF3.1).

When we mis-specified the models by missing one of the two confounders or the quadratic term, the biases, standardized biases, 95% coverage probabilities and MSEs were in the reasonable range in general (Tables AF3.1-AF3.4). However, when we ignored the interaction term, the estimates were quite poor. For example, the relative biases were in the range of 47-59% when the effect modifier was moderately associated with the outcome and greater than 120% when the effect modifier was strongly associated with the outcome variable (Table AF3.1). As another example, the 95% coverage probabilities dropped to 70%-80% range when the effect modifier was moderately associated with the outcome and to 20%-40% range when the effect modifier was strongly associated with the outcome variable, when the outcome rate was 10% (Table AF3.3). The 95% coverage probabilities were worse when the outcome=25%.

The relative biases, the standardized biases and the MSEs seemed to increase with RR, except for scenario c1 and outcome=25% in which the relative biases did not reveal any particular pattern (Tables AF3.1, AF3.2 and AF3.4). However, the increase seemed to be more dramatic when the outcome was 10%.

**Conclusions**

The findings suggested that the two models performed almost identically in all scenarios we studied, regardless of the outcome rate, the risk ratio and the strength of association between the confounder and the exposure (or the outcome).

**References**

1. SAS *Software Version 9.2* of the SAS System for Unix. SAS Institute Inc. 2008. Cary, NC.
2. Deddens JA, Petersen MR, Lei X. *Estimation of Prevalence Ratios When PROC GENMOD Does not Converge.* SAS Users Group International Proceedings, (SUGI28) 2003. Seattle, Washington.
3. Petersen MR, Deddens JA. A revised SAS macro for maximum likelihood estimation of prevalence ratios using the COPY method [letter]. *Occupational and Environmental Medicine.* 2009; 66(9) , p. 639, DOI: [10.1136/oem.2008.043018](http://dx.doi.org/10.1136/oem.2008.043018).

Table AF3.1. Percentage Bias (%) of Risk Ratio by Type of Misspecification, Estimation Method and Outcome Rate

| Type of Misspecification | | | Outcome Rate | | | | | |
| --- | --- | --- | --- | --- | --- | --- | --- | --- |
|  |  |  | 10% | | | 25% | | |
|  |  |  | Risk Ratio | | | Risk Ratio | | |
|  |  |  | 1.0 | 1.5 | 2.0 | 1.0 | 1.5 | 2.0 |
| Ignoring one of the two confounders | a1 | log binomial | 4.8 | 5.3 | 6.6 | 2.3 | 3.0 | 3.2 |
|  |  | Robust Poisson | 4.8 | 5.3 | 6.6 | 2.3 | 3.0 | 3.2 |
|  | a2 | log binomial | 6.8 | 7.3 | 9.0 | 3.4 | 3.9 | 3.9 |
|  |  | Robust Poisson | 6.8 | 7.3 | 9.0 | 3.4 | 3.9 | 3.9 |
|  | a3 | log binomial | 5.4 | 6.8 | 9.5 | 3.2 | 5.0 | 4.1 |
|  |  | Robust Poisson | 5.4 | 6.8 | 9.5 | 3.2 | 5.0 | 4.1 |
|  | a4 | log binomial | 8.5 | 9.4 | 11.2 | 5.3 | 5.2 | 6.2 |
|  |  | Robust Poisson | 8.5 | 9.4 | 11.2 | 5.3 | 5.2 | 6.2 |
| Ignoring a quadratic term | b1 | log binomial | 5.1 | 6.0 | 6.1 | 1.1 | 1.9 | 2.5 |
|  |  | Robust Poisson | 5.0 | 6.0 | 6.1 | 1.2 | 1.9 | 2.5 |
|  | b2 | log binomial | 4.6 | 5.8 | 7.1 | 1.2 | 1.4 | 2.1 |
|  |  | Robust Poisson | 4.6 | 5.9 | 7.1 | 1.2 | 1.4 | 2.1 |
|  | b3 | log binomial | 5.7 | 4.8 | 7.5 | 0.9 | 1.3 | 2.8 |
|  |  | Robust Poisson | 5.7 | 4.8 | 7.5 | 0.8 | 1.3 | 2.7 |
|  | b4 | log binomial | 3.0 | 4.9 | 6.5 | 1.9 | 1.7 | 1.7 |
|  |  | Robust Poisson | 3.0 | 4.9 | 6.5 | 1.9 | 1.7 | 1.8 |
| Ignoring an interaction term | c1 | log binomial | 50.8 | 55.5 | 59.4 | 47.7 | 49.3 | 48.5 |
|  |  | Robust Poisson | 50.7 | 55.4 | 59.2 | 47.3 | 48.9 | 48.2 |
|  | c2 | log binomial | 127.6 | 139.1 | 140.6 | 122.0 | 122.0 | 126.1 |
|  |  | Robust Poisson | 126.9 | 138.6 | 140.1 | 120.0 | 120.3 | 124.6 |

Table AF3.2. Standardized Bias (%) of Risk Ratio by Type of Misspecification, Estimation Method and Outcome Rate

| Type of Misspecification | | | Outcome Rate | | | | | |
| --- | --- | --- | --- | --- | --- | --- | --- | --- |
|  |  |  | 10% | | | 25% | | |
|  |  |  | Risk Ratio | | | Risk Ratio | | |
|  |  |  | 1.0 | 1.5 | 2.0 | 1.0 | 1.5 | 2.0 |
| Ignoring one of the two confounders | a1 | log binomial | 17.48 | 28.29 | 44.81 | 14.49 | 28.21 | 37.95 |
|  |  | Robust Poisson | 17.51 | 28.22 | 44.79 | 14.53 | 28.12 | 38.05 |
|  | a2 | log binomial | 25.05 | 38.73 | 61.03 | 21.75 | 36.65 | 45.91 |
|  |  | Robust Poisson | 24.96 | 38.64 | 61.12 | 22.03 | 36.47 | 45.79 |
|  | a3 | log binomial | 19.62 | 36.20 | 64.07 | 20.55 | 46.07 | 48.23 |
|  |  | Robust Poisson | 19.61 | 36.22 | 64.07 | 20.50 | 45.95 | 48.20 |
|  | a4 | log binomial | 31.20 | 49.98 | 75.00 | 34.23 | 48.11 | 72.31 |
|  |  | Robust Poisson | 31.11 | 49.96 | 75.04 | 34.16 | 47.99 | 72.22 |
| Ignoring a quadratic term | b1 | log binomial | 18.55 | 31.94 | 41.57 | 7.49 | 17.86 | 30.03 |
|  |  | Robust Poisson | 18.17 | 31.85 | 41.43 | 7.94 | 18.33 | 29.26 |
|  | b2 | log binomial | 16.80 | 30.97 | 48.00 | 7.93 | 12.73 | 24.93 |
|  |  | Robust Poisson | 16.80 | 31.08 | 48.06 | 7.80 | 12.96 | 24.87 |
|  | b3 | log binomial | 20.87 | 25.60 | 50.20 | 5.63 | 12.50 | 32.41 |
|  |  | Robust Poisson | 20.86 | 25.42 | 50.17 | 5.05 | 12.05 | 31.78 |
|  | b4 | log binomial | 10.94 | 26.31 | 44.19 | 11.91 | 15.85 | 20.58 |
|  |  | Robust Poisson | 10.95 | 26.23 | 44.13 | 11.90 | 15.86 | 20.75 |
| Ignoring an interaction term | c1 | log binomial | 182.63 | 279.36 | 369.35 | 299.84 | 430.27 | 522.69 |
|  |  | Robust Poisson | 182.17 | 278.55 | 368.50 | 296.92 | 426.31 | 518.46 |
|  | c2 | log binomial | 430.92 | 629.46 | 776.91 | 721.67 | 962.56 | 1190.40 |
|  |  | Robust Poisson | 428.30 | 626.65 | 773.55 | 705.68 | 944.77 | 1172.10 |

Table AF3.3. Empirical Coverage of 95% Two-Sided Confidence Interval of Risk Ratio by Type, Misspecification, Estimation Method and Outcome Rate

| Type of Misclassification | | | Outcome Rate | | | | | |
| --- | --- | --- | --- | --- | --- | --- | --- | --- |
|  |  |  | 10% | | | 25% | | |
|  |  |  | Risk Ratio | | | Risk Ratio | | |
|  |  |  | 1.0 | 1.5 | 2.0 | 1.0 | 1.5 | 2.0 |
| Ignoring one of the two confounders | a1 | log binomial | 95.6 | 96.0 | 95.7 | 95.5 | 95.5 | 96.2 |
|  |  | Robust Poisson | 95.6 | 95.9 | 95.7 | 95.3 | 95.5 | 96.3 |
|  | a2 | log binomial | 94.8 | 95.3 | 95.9 | 94.4 | 94.6 | 95.1 |
|  |  | Robust Poisson | 95.0 | 95.3 | 95.8 | 94.4 | 94.6 | 95.3 |
|  | a3 | log binomial | 95.6 | 95.2 | 95.3 | 94.6 | 94.4 | 95.3 |
|  |  | Robust Poisson | 95.6 | 95.3 | 95.6 | 94.8 | 94.3 | 95.3 |
|  | a4 | log binomial | 95.0 | 94.8 | 95.2 | 95.0 | 95.4 | 95.3 |
|  |  | Robust Poisson | 95.1 | 94.8 | 95.1 | 95.1 | 95.2 | 95.2 |
| Ignoring a quadratic term | b1 | log binomial | 95.5 | 95.1 | 95.2 | 95.0 | 95.8 | 95.3 |
|  |  | Robust Poisson | 95.4 | 95.0 | 95.3 | 94.9 | 95.4 | 94.7 |
|  | b2 | log binomial | 95.1 | 95.8 | 96.0 | 95.3 | 94.5 | 95.7 |
|  |  | Robust Poisson | 95.0 | 95.8 | 96.0 | 95.2 | 94.5 | 95.8 |
|  | b3 | log binomial | 95.1 | 96.2 | 95.9 | 95.5 | 96.0 | 94.6 |
|  |  | Robust Poisson | 95.0 | 96.1 | 95.7 | 95.1 | 96.0 | 94.3 |
|  | b4 | log binomial | 94.9 | 95.8 | 95.7 | 95.3 | 95.0 | 95.4 |
|  |  | Robust Poisson | 95.0 | 95.7 | 95.8 | 95.2 | 95.1 | 95.5 |
| Ignoring an interaction term | c1 | log binomial | 73.7 | 76.5 | 79.0 | 32.2 | 38.4 | 47.7 |
|  |  | Robust Poisson | 73.9 | 76.7 | 79.0 | 32.7 | 38.8 | 48.5 |
|  | c2 | log binomial | 22.0 | 29.5 | 38.8 | 0.0 | 0.4 | 1.1 |
|  |  | Robust Poisson | 22.6 | 29.8 | 39.5 | 0.2 | 0.5 | 1.3 |

Table AF3.4. Mean Square Error of Risk Ratio by Type of Misspecification, Estimation Method and Outcome Rate

| Type of Misspecification | | | Outcome Rate | | | | | |
| --- | --- | --- | --- | --- | --- | --- | --- | --- |
|  |  |  | 10% | | | 25% | | |
|  |  |  | Risk Ratio | | | Risk Ratio | | |
|  |  |  | 1.0 | 1.5 | 2.0 | 1.0 | 1.5 | 2.0 |
| Ignoring one of the two confounders | a1 | log binomial | 0.0769 | 0.0851 | 0.1038 | 0.0248 | 0.0279 | 0.0328 |
|  |  | Robust Poisson | 0.0770 | 0.0850 | 0.1038 | 0.0248 | 0.0279 | 0.0329 |
|  | a2 | log binomial | 0.0793 | 0.0913 | 0.1198 | 0.0252 | 0.0293 | 0.0348 |
|  |  | Robust Poisson | 0.0792 | 0.0913 | 0.1198 | 0.0253 | 0.0293 | 0.0348 |
|  | a3 | log binomial | 0.0784 | 0.0903 | 0.1241 | 0.0254 | 0.0318 | 0.0359 |
|  |  | Robust Poisson | 0.0784 | 0.0903 | 0.1241 | 0.0255 | 0.0318 | 0.0359 |
|  | a4 | log binomial | 0.0822 | 0.1001 | 0.1384 | 0.0271 | 0.0322 | 0.0447 |
|  |  | Robust Poisson | 0.0822 | 0.1000 | 0.1384 | 0.0272 | 0.0322 | 0.0447 |
| Ignoring a quadratic term | b1 | log binomial | 0.0767 | 0.0864 | 0.1011 | 0.0234 | 0.0259 | 0.0307 |
|  |  | Robust Poisson | 0.0767 | 0.0864 | 0.1010 | 0.0237 | 0.0261 | 0.0308 |
|  | b2 | log binomial | 0.0774 | 0.0877 | 0.1080 | 0.0245 | 0.0264 | 0.0307 |
|  |  | Robust Poisson | 0.0774 | 0.0878 | 0.1080 | 0.0246 | 0.0265 | 0.0307 |
|  | b3 | Log binomial | 0.0782 | 0.0844 | 0.1107 | 0.0236 | 0.0257 | 0.0318 |
|  |  | Robust Poisson | 0.0782 | 0.0843 | 0.1107 | 0.0239 | 0.0259 | 0.0319 |
|  | b4 | log binomial | 0.0755 | 0.0843 | 0.1034 | 0.0245 | 0.0264 | 0.0298 |
|  |  | Robust Poisson | 0.0755 | 0.0843 | 0.1034 | 0.0246 | 0.0264 | 0.0299 |
| Ignoring an interaction term | c1 | log binomial | 0.3354 | 0.7821 | 1.5125 | 0.2528 | 0.5769 | 0.9769 |
|  |  | Robust Poisson | 0.3342 | 0.7787 | 1.5066 | 0.2490 | 0.5685 | 0.9641 |
|  | c2 | log binomial | 1.7151 | 4.4662 | 8.0343 | 1.5180 | 3.3860 | 6.4034 |
|  |  | Robust Poisson | 1.6981 | 4.4334 | 7.9790 | 1.4687 | 3.2945 | 6.2595 |
