# Supplementary material for: Comparing performance between log-binomial and robust Poisson regression models for estimating risk ratios under model misspecification
Source: BMC Med Res Methodol. 2018 Jun 22;18:63. doi: 10.1186/s12874-018-0519-5 (PMC6013902; doi:10.1186/s12874-018-0519-5)
Supplement: Supplementary file 4 — Using Fish scoring (iteration) to estimate β. Proof of the iteration equation in Methods section. (DOCX 45 kb) [file 12874_2018_519_MOESM4_ESM.docx]

**Additional file 4. Using Fish scoring (iteration) to estimate**$\beta$**. Proof of the iteration equation in Methods section.**

Maximum likelihood estimation of the log-binomial model is

$=\sum_{i=1}^{n} \left(\frac{y_{i}}{p_{i}(\beta)}-\frac{(1-y_{i})}{(1-p_{i}(\beta))}\right) \cdot\frac{d(p_{i}(\beta))}{d\beta_{j}}$

$=\sum_{i=1}^{n} \frac{(y_{i}-p_{i}(\beta))}{p_{i}(\beta)\cdot(1-p_{i}(\beta))}\cdot\frac{d(p_{i}(\beta))}{d\beta_{j}}$.

Since$p_{i}(\beta)=\exp(\text{x}_{i}^{'}\beta)=\exp(\beta_{0}+\sum_{j=1}^{k} x_{ij}\beta_{j})$,

$\frac{d(p_{i}(\beta))}{d\beta_{j}}=\exp(\beta_{0}+\sum_{i=1}^{k} x_{ij}\beta_{j})\cdot x_{ij}=p_{i}(\beta)\cdot x_{ij}$, and thus

$=\sum_{i=1}^{n} \frac{(y_{i}-p_{i}(\beta))}{(1-p_{i}(\beta))}\cdot x_{ij}$, where j=1,..,K. (1)

To find the expected second derivatives, we can first rearrange the 1^st^ derivatives derived above.

$=\sum_{i=1}^{n} \frac{y_{i}-1}{1-p_{i}(\beta)} \cdot x_{ij}+\sum_{i=1}^{n} x_{ij}$, and

$=\sum_{i=1}^{n} (y_{i}-1)\cdot x_{ij}\cdot\frac{1}{(1-p_{i}(\beta))^{2}} \cdot\frac{d(p_{i}(\beta))}{d\beta_{l}}$.

Since$\frac{d(p_{i}(\beta))}{d\beta_{l}}=\exp(\beta_{0}+\sum_{l=1}^{k} x_{il}\beta_{l})\cdot x_{il}=p_{i}(\beta)\cdot x_{il}$,

,

and thus , where 1≤j, l≤K (2)

According to Fisher scoring, the MLE for$\beta$can be carried out by the following iteration.

, where is the log-likelihood function for the entire sample and () is the first (second) derivative. (3)

Rearranging equation (3) gives

. (4)

In the matrix form, and, where$X=(x_{i,j})\in R^{n,k}$, $\frac{Y-P(\beta)}{P(\beta)}=\left(\frac{y_{1}-p_{1}(\beta)}{p_{1}(\beta)},\frac{y_{2}-p_{2}(\beta)}{p_{2}(\beta)},...,\frac{y_{n}-p_{n}(\beta)}{p_{n}(\beta)}\right)^{T}$, and W (referred to as weight) =.$Diag(\frac{p_{i}(\beta)}{1-p_{i}(\beta)})$, i=1,2,…,n; j=1,2,…,k. (5)

Plugging (5) into (4), equation (4) above becomes$\beta^{(t+1)}=(X'WX)^{-1}(X'Wz)$, where $z=X\beta^{(t)}+\frac{(Y-P(\beta^{(t)}))}{P(\beta^{(t)})}$. (6)
