# Supplementary material for: Comparing performance between log-binomial and robust Poisson regression models for estimating risk ratios under model misspecification
Source: BMC Med Res Methodol. 2018 Jun 22;18:63. doi: 10.1186/s12874-018-0519-5 (PMC6013902; doi:10.1186/s12874-018-0519-5)
Supplement: Supplementary file 5 — SAS codes used to estimate β for each regression model. (DOCX 12 kb) [file 12874_2018_519_MOESM5_ESM.docx]

Additional file 5. SAS codes used to estimate β for each regression model

Log-binomial regression model

Proc GENMOD data=datasetname descending;

Model y = x z_1_ z_2_ /dist=binomial link=log intercept=-**4**;

Output out=preds p=pred l=lower u=upper;

Run;

Robust Poisson regression model

Proc GENMOD data=datasetname;

Class subj_id ;

Model y = x z_1_ z_2_ / dist=poisson link=log;

Repeated subject=subj_id/type=unstr;

Output out=preds p=pred l=lower u=upper;

Run;
