# Supplementary material for: Comparing performance between log-binomial and robust Poisson regression models for estimating risk ratios under model misspecification
Source: BMC Med Res Methodol. 2018 Jun 22;18:63. doi: 10.1186/s12874-018-0519-5 (PMC6013902; doi:10.1186/s12874-018-0519-5)
Supplement: Supplementary file 6 — Results on simulated data when n = 500. (DOCX 18 kb) [file 12874_2018_519_MOESM6_ESM.docx]

**Additional file 6. Results on simulated data when n=500**

Unshaded: Models were correctly specified

Light shaded: Mis-specified linear predictors or mis-specified link function

Dark shaded: Mis-specified linear predictors and mis-specified link function

LB: log-binomial, RP: robust Poisson

Table AF6.1. Relative bias (%) in log scale with and without model misspecification (n=500)

| Scenario | 1 | | 2 | | 3 | | 4 | |
| --- | --- | --- | --- | --- | --- | --- | --- | --- |
|  | LB | RP | LB | RP | LB | RP | LB | RP |
| I- | 1.4 | 1.8 | 2.0 | 2.5 | -0.7 | 1.0 | -3.5 | 1.6 |
| II- | 1.6 | 1.6 | 0.9 | 2.1 | -1.9 | 1.9 | -5.8 | 2.1 |
| III- | 1.7 | 2.3 | -2.2 | 0.7 | -4.2 | 1.7 | -10.8 | 0.9 |
|  |  |  |  |  |  |  |  |  |
| IV- | 0.3 | 0.9 | -1.1 | 0.7 | -2.7 | 1.0 | -5.7 | 1.2 |
| III- | 1.7 | 2.3 | -2.2 | 0.7 | -4.2 | 1.7 | -10.8 | 0.9 |
| V- | 1.2 | 2.0 | -2.9 | 1.1 | -6.0 | 2.4 | -13.4 | 2.1 |
|  |  |  |  |  |  |  |  |  |
| III- | 1.7 | 2.3 | -2.2 | 0.7 | -4.2 | 1.7 | -10.8 | 0.9 |
| VI- | -1.4 | 1.1 | -4.3 | 0.2 | -5.5 | 2.0 | -10.6 | 0.9 |
| VII- | -5.1 | 2.0 | -10.0 | 1.9 | -15.4 | 1.2 | -19.8 | 1.8 |

Change of scenarios: Increasing intercept: I →II→III; Increasing coefficient of β_2_: IV→III→V; Change of link function: III (log), VI (logit), VII (probit).

Table AF6.2 Standard error in log scale with and without model misspecification (n=500)

| Scenario | 1 | | 2 | | 3 | | 4 | |
| --- | --- | --- | --- | --- | --- | --- | --- | --- |
|  | LB | RP | LB | RP | LB | RP | LB | RP |
| I- | 0.30 | 0.30 | 0.26 | 0.27 | 0.24 | 0.24 | 0.20 | 0.21 |
| II- | 0.26 | 0.27 | 0.23 | 0.24 | 0.22 | 0.22 | 0.18 | 0.19 |
| III- | 0.24 | 0.25 | 0.21 | 0.23 | 0.20 | 0.21 | 0.17 | 0.18 |
|  |  |  |  |  |  |  |  |  |
| IV- | 0.19 | 0.20 | 0.18 | 0.19 | 0.17 | 0.19 | 0.16 | 0.17 |
| III- | 0.24 | 0.25 | 0.21 | 0.23 | 0.20 | 0.21 | 0.17 | 0.18 |
| V- | 0.26 | 0.27 | 0.22 | 0.24 | 0.22 | 0.24 | 0.18 | 0.20 |
|  |  |  |  |  |  |  |  |  |
| III- | 0.24 | 0.25 | 0.21 | 0.23 | 0.20 | 0.21 | 0.17 | 0.18 |
| VI- | 0.20 | 0.22 | 0.19 | 0.20 | 0.18 | 0.19 | 0.16 | 0.17 |
| VII- | 0.30 | 0.32 | 0.26 | 0.28 | 0.22 | 0.25 | 0.20 | 0.21 |

Change of scenarios: Increasing intercept: I →II→III; Increasing coefficient of β_2_: IV→III→V; Change of link function: III (log), VI (logit), VII (probit).

Table AF6.3. Mean square error (MSE) in log scale with and without model misspecification (n=500)

| Scenario | 1 | | 2 | | | 3 | | | 4 | |
| --- | --- | --- | --- | --- | --- | --- | --- | --- | --- | --- |
|  | LB | RP | | LB | RP | | LB | RP | LB | RP |
| I- | 0.090 | 0.093 | | 0.069 | 0.072 | | 0.056 | 0.060 | 0.041 | 0.045 |
| II- | 0.066 | 0.071 | | 0.054 | 0.060 | | 0.047 | 0.051 | 0.038 | 0.038 |
| III- | 0.057 | 0.064 | | 0.046 | 0.053 | | 0.042 | 0.046 | 0.043 | 0.033 |
|  |  |  | |  |  | |  |  |  |  |
| IV- | 0.036 | 0.039 | | 0.033 | 0.036 | | 0.031 | 0.034 | 0.029 | 0.027 |
| III- | 0.057 | 0.064 | | 0.046 | 0.053 | | 0.042 | 0.046 | 0.043 | 0.033 |
| V- | 0.068 | 0.075 | | 0.049 | 0.056 | | 0.053 | 0.056 | 0.056 | 0.039 |
|  |  |  | |  |  | |  |  |  |  |
| III- | 0.057 | 0.064 | | 0.046 | 0.053 | | 0.042 | 0.046 | 0.043 | 0.033 |
| VI- | 0.042 | 0.047 | | 0.038 | 0.040 | | 0.034 | 0.036 | 0.041 | 0.030 |
| VII- | 0.092 | 0.106 | | 0.079 | 0.082 | | 0.078 | 0.061 | 0.086 | 0.045 |

Change of scenarios: Increasing intercept: I →II→III; Increasing coefficient of β_2_: IV→III→V; Change of link function: III (log), VI (logit), VII (probit).
